# The microRNAs as potential biomarkers for predicting the onset of aflatoxin exposure in human beings: a review

**DOI:** 10.3389/fmicb.2014.00102

**Published:** 2014-03-18

**Authors:** Rafael Valencia-Quintana, Juana Sánchez-Alarcón, María G. Tenorio-Arvide, Youjun Deng, José M. R. Montiel-González, Sandra Gómez-Arroyo, Rafael Villalobos-Pietrini, Josefina Cortés-Eslava, Ana R. Flores-Márquez, Francisco Arenas-Huertero

**Affiliations:** ^1^Evaluación de Riesgos Ambientales, Facultad de Agrobiología, Universidad Autónoma de TlaxcalaTlaxcala, México; ^2^Departamento de Investigación en Ciencias Agrícolas, Benemérita Universidad Autónoma de PueblaPuebla, México; ^3^Department of Soil and Crop Sciences, Texas AgriLife, Texas A&M UniversityCollege Station, TX, USA; ^4^Departamento de Ciencias Ambientales, Centro de Ciencias de la Atmósfera, Universidad Nacional Autónoma de MéxicoDistrito Federal, México; ^5^Laboratorio de Patología Experimental, Hospital Infantil de México Federico GómezDistrito Federal, México

**Keywords:** AFB1, aflatoxin exposure, microRNAs, HCC, potential biomarkers

## Abstract

The identification of aflatoxins as human carcinogens has stimulated extensive research efforts, which continue to the present, to assess potential health hazards resulting from contamination of the human food supply and to minimize exposure. The use of biomarkers that are mechanistically supported by toxicological studies will be important tools for identifying stages in the progression of development of the health effects of environmental agents. miRNAs are small non-coding mRNAs that regulate post-transcriptional gene expression. Also, they are molecular markers of cellular responses to various chemical agents. Growing evidence has demonstrated that environmental chemicals can induce changes in miRNA expression. miRNAs are good biomarkers because they are well defined, chemically uniform, restricted to a manageable number of species, and stable in cells and in the circulation. miRNAs have been used as serological markers of HCC and other tumors. The expression patterns of different miRNAs can distinguish among HCC-hepatitis viruses related, HCC cirrhosis-derivate, and HCC unrelated to either of them. The main objective of this review is to find unreported miRNAs in HCC related to other causes, so that they can be used as specific molecular biomarkers in populations exposed to aflatoxins and as early markers of exposure, damage/presence of HCC. Until today specific miRNAs as markers for aflatoxins-exposure and their reliability are currently lacking. Based on their elucidated mechanisms of action, potential miRNAs that could serve as possible markers of HCC by exposure to aflatoxins are miR-27a, miR-27b, miR-122, miR-148, miR-155, miR-192, miR-214, miR-221, miR-429, and miR-500. Future validation for all of these miRNAs will be needed to assess their prognostic significance and confirm their relationship with the induction of HCC due to aflatoxin exposure.

## Introduction

The aflatoxins were structurally identified in the early 1960s and over the last 50 years have been extensively studied with respect to their mechanisms of action, including their mutagenic and carcinogenic activity. This work has been paralleled by developments in biomarkers of aflatoxin metabolism, DNA adducts, and mutations applied to exposed human populations. The improvements in exposure assessment in epidemiological studies and the demonstration of a specific mutation in the *TP53* gene have contributed significantly to the identification of aflatoxins as human carcinogens. In addition, the studies of animal and human aflatoxin metabolism have provided opportunities to develop chemoprevention approaches in human populations (Wild and Turner, 2002; Valencia-Quintana et al., 2012). These findings stimulated extensive research efforts, which continue to the present, to assess potential health hazards resulting from contamination of the human food supply and to minimize exposure (Kensler et al., 2011).

The use of biomarkers that are mechanistically supported by toxicological studies will be important tools for identifying stages in the progression of development of the health effects of environmental agents. Since the development of a general paradigm for molecular epidemiology and biomarkers nearly 20 years ago, progress has been made in applying these tools to specific environmental situations that may be hazardous to humans, as exemplified by AFB1 studies. The major goals of molecular epidemiology research are to develop and to validate biomarkers that reflect specific exposures, their interactions, and predictions of disease risk in individuals. Presumably, after an environmental exposure each person has a unique response to both dose and time to disease onset. These responses will be affected both by genetic, host and environmental modifiers. It is assumed that biomarkers that reflect the mechanisms of action of the etiologic agents will be strong predictors of an individual's risk of disease. It is also expected that these biomarkers can more clearly classify the status of exposure of individuals and general populations (Groopman et al., 2005).

Biomarkers can be used as outcome measures in these and primary prevention studies. Overall, the integrated, multidisciplinary research on aflatoxins has provided the scientific platform on which to base decisions regarding acceptable exposures and priorities for interventions to reduce human risk in a public health context (Wild and Turner, 2002).

## Aflatoxin biomarkers

AFB1 requires metabolic activation to its ultimate carcinogenic form, a reactive epoxide (aflatoxin-8,9-epoxide), primarily by the cytochrome P450 (CYP) monooxygenase system. Epoxidation is catalyzed by CYP1A2 and CYP3A4 in humans (Gallagher et al., 1994; Ueng et al., 1995). Many other oxidation products, including aflatoxin M1, are also formed. The epoxide can react further by interacting with DNA to produce a promutagenic aflatoxin-N7-guanine adduct. This adduct is unstable in DNA, rapidly undergoes depurination, and is excreted in urine (Bennett et al., 1981). The epoxide can also form products that react with serum albumin to form long-lived lysine adducts (Sabbioni et al., 1987). In addition, the epoxide can be conjugated by certain glutathione S-transferases (GSTs), which are further metabolized to form aflatoxin-mercapturic acid detoxification products that can be excreted in urine (Scholl et al., 1997). Urinary measures of aflatoxin M1, aflatoxin-mercapturic acid, and the aflatoxin-albumin adduct are used as biomarkers of internal dose. Aflatoxin-N7-guanine in urine serves as an elegant biomarker of biologically effective dose because it is clear that formation of this adduct lies on the causal pathway to aflatoxin-induced HCC (Kensler et al., 2011).

An objective in development of AFB1 biomarkers is to use them as predictors of past and future exposure status in people (Kensler et al., 2011). However, two key attributes, one biological (tracking) and the other chemical (stability), need to be confirmed to successfully use biomarkers for these purposes. miRNAs are good biomarkers because they are well defined, chemically uniform, restricted to a manageable number of species, and stable in cells and in the circulation (Wang et al., 2012a).

## microRNAs and environmental pollutants

Exposure to environmental chemicals is well known to increase risks for various diseases (Crinnion, 2010; Newbold, 2010), and gene expression can be changed as a response to these exogenous stressors (Ueda, 2009; Patel and Butte, 2010; Hou et al., 2012), like tobacco and polycyclic aromatic hydrocarbons in urban air of megacities (Arenas-Huertero et al., 2011). Such changes may be regulated by specific miRNAs and emerged as a gene expression regulatory factor that may link environmental chemicals and their related diseases.

Secreted miRNAs have many requisite features of good biomarkers. miRNAs are stable in various bodily fluids, the sequences of most miRNAs are conserved among different species, the expression of some miRNAs is specific to tissues or biological stages, and the level of miRNAs can be easily assessed by various methods, as polymerase chain reaction (PCR), which allows for signal amplification. The changes of several miRNA levels in plasma, serum, urine, and saliva have already been associated with different diseases (for review see Etheridge et al., 2011).

Growing evidence has demonstrated that environmental chemicals can induce changes in miRNA expression (Hou et al., 2011). Arsenite exposure induced significant decrease in miR-19a expression in human lymphoblast cells line TK-6, resulting in cell growth arrest and apoptosis (Marsit et al., 2006). Metal sulfates have been shown to generate reactive oxygen species (ROS) and trigger the expression of specific miRNAs (Lukiw and Pogue, 2007). Bollati et al. (2010) found an increased expression of miR-146a related to inhalation of Cd-rich air particles in steel workers, and induced rapid changes in the expression of two inflammation-related miRNAs, miR-21 and miR-222. Aluminum exposure may induce genotoxicity via miRNA-related regulatory elements, for example, miR-146a, miR-9, miR-125b, and miR-128 (Lukiw and Pogue, 2007; Pogue et al., 2009).

Jardim et al. (2009) have shown extensive alterations of miRNA expression profiles in human bronchial epithelial cells treated with diesel exhaust particles. Schembri et al. (2009) have identified 28 miRNAs that were differentially expressed in smokers when compared to non-smokers, changes in miRNA expression were suggested to contribute to altered regulation of oncogenes, tumor suppressor genes, oxidative stress, xenobiotic metabolism, and inflammation. Izzotti et al. (2009a,b) have monitored the expression of 484 miRNAs in the lungs of mice exposed to cigarette smoking, the most remarkably downregulated miRNAs belonged to several miRNA families, such as let-7, miR-10, miR-26, miR-30, miR-34, miR-99, miR-122, miR-123, miR-124, miR-125, miR-140, miR-145, miR-146, miR-191, miR-192, miR-219, miR-222, and miR-223. These miRNAs regulate expression of genes involved in stress responses, apoptosis, proliferation, and angiogenesis.

Zhang and Pan (2009) have evaluated the effects of Hexahydro-1, 3, 5-trinitro-1, 3, 5-triazine (also known as hexogen or cyclonite) (RDX) on miRNA expression in mouse brain and liver, most of the miRNAs that showed altered expression, including let-7, miR-17-92, miR-10b, miR-15, miR-16, miR-26, and miR-181, were related to toxicant-metabolizing enzymes, and genes related to carcinogenesis, and neurotoxicity, in addition, consistent with the known neurotoxic effects of RDX, the authors documented significant changes in miRNA expression in the brains of RDX-treated animals, such as miR-206, miR-30a, miR-30c, miR-30d, and miR-195. STS (sodium thiosulfate) treatment also resulted in differential expression of miR-124a and miR-133a in the treated embryos (Choudhuri, 2010). Fukushima et al. (2007) have demonstrated that rat exposed to acetaminophen or carbon tetrachloride showed down-regulation of miR-298 and miR-370 in the liver that was accompanied by hepatocyte necrosis and inflammation. Wang et al. (2009a) found increase serum concentration of hepatocyte-specific miRNAs including miR-122 and miR-192 within 1 h after acetaminophen exposure. In mouse exposure to Wy-14,643, peroxisome proliferator-activated receptor alpha (PPARα) agonist, up-regulate let-7C (Shah et al., 2007). Ethanol exposure down-regulate miR-21, miR-335, miR-9, and miR-153 (Sathyan et al., 2007). In rats, tamoxifen up-regulate miR-17-92 cluster, miR-106a, and miR-34 (Pogribny et al., 2007). 4-(methylnitrosamino)-1-(3-pyridyl)-1-butanone (NNK), a tobacco carcinogen, down-regulate miR-34, miR-101, miR-126, and miR-199 (Kalscheuer et al., 2008). In humans, 1 alpha, 25-dihydroxyvitamin D(3) [1,25(OH)(2)D(3)], major vitamin D metabolite, up-regulate miR-125b (Mohri et al., 2009), and 5-fluorouracil (5-FU) an antineoplasic drug, up-regulate miR-200b (Rossi et al., 2007). Bisphenol A (Avissar-Whiting et al., 2010), dioxin (Elyakim et al., 2010), and diethylstilbestrol (DES) (Hsu et al., 2009) desregulate expression of miR-146a, miR-191, and miR-9-3, respectively. Moffat et al. (2007) looked at the effects of dioxin treatment on miRNA in mice, dioxin-resistant rats (Han/Wistar; Kuopio) and dioxin-sensitive rats (Long–Evans; Turku/AB), it is interesting to note that the dioxin sensitive rats had more affected miRNAs. X-ray exposure resulted in down regulation of miR-7 (Ilnytskyy et al., 2008). The ROS induction resulted in up-regulation of a specific set of miRNAs, including miR-9, miR-125b, and miR-128 (Lukiw and Pogue, 2007). Lin et al. (2009), and Cheng et al. (2009), found that hydrogen peroxide induce up-regulation of miR-21.

The increasing evidence that the expression of miRNAs is affected by several known toxicants as well as oxidative and other forms of cellular stress certainly suggest an important role of miRNAs in toxicology, which could provide a link between environmental influences and gene expression (Lema and Cunningham, 2010). Analysis of the resulting molecular signatures provides new tools to identify mechanisms of toxicity, as well as to classify compounds based on the biological response they elicit and to identify cluster of genes detective or predictive of certain type of toxic response, which are employed as biomarkers (Gatzidou et al., 2007). miRNAs are one of the main mechanisms of epigenetic regulation of gene expression. Conversely to stress-related miRNAs, the toxicological research of miRNAs associated to specific toxicants has started few years ago. Therefore, available publications are focused in a broad spectra of toxicants and compiled research about a particular miRNA is yet very limited (Lema and Cunningham, 2010).

## microRNA in HCC as potential biomarkers of aflatoxin exposure

In the recent years, several studies revealed that the expression of miRNAs is deregulated in human HCC in comparison with matched non-neoplastic tissue (Lu et al., 2005; Gramantieri et al., 2008; Law and Wong, 2011; Borel et al., 2012a; Wang et al., 2012a,b; Sun and Karin, 2013; Wong et al., 2013). Some miRNAs identified in HCC are reported in Table 1.

**Table 1:** **microRNA deregulated in HCC**.

**miR-**	**Effects over**	**References**
miR-9/9^*^/-2	Promote HCC migration and invasion through regulation of KLF17	Budhu et al., 2008; Wang et al., 2008; Sun et al., 2013; Xu et al., 2013
miR-10b	Promoted cell migration and invasion	Ladeiro et al., 2008; Li et al., 2010
miR-15b	Molecular mechanisms and roles in HCC remain largely unknown	Liu et al., 2012; Wong et al., 2013
miR-17/-5p	Proliferation and migration	Kutay et al., 2006; Huang et al., 2009; Yang et al., 2010; Chen et al., 2012a; Zheng et al., 2013
miR-17-92	Induce proliferation and anchorage-independent growth	Pogribny et al., 2007; Wang et al., 2012a
miR-18/a/p-18	High expression in HCC tumors. Promote cell growth. Proliferation	Murakami et al., 2006; Jiang et al., 2008; Liu et al., 2009; Kerr et al., 2011
miR-19a	Proliferation	Budhu et al., 2008; Connolly et al., 2008; Wong et al., 2008; Li et al., 2009c
miR-20/a	Proliferation, recurrence, and prognosis	Kutay et al., 2006; Fan et al., 2013
miR-21	Modulating PTEN expression and PTEN-dependent pathways. Enhanced AKT pathway. Promote cell cycle progression, reduce cell death and favor angiogenesis and invasion. Able to differentiate HCC from chronic hepatitis	Kutay et al., 2006; Volinia et al., 2006; Meng et al., 2007; Gramantieri et al., 2008; Huang et al., 2008; Jiang et al., 2008; Ladeiro et al., 2008; Wang et al., 2008; Chen, 2009; Garofalo et al., 2009; Li et al., 2009b; Pogribny et al., 2009; Pineau et al., 2010; Wong et al., 2010, 2013; Xu et al., 2011; Tomimaru et al., 2012; Karakatsanis et al., 2013
miR-22	Enhanced NF-kB signaling	Takata et al., 2011
miR-23a/b	Repress the expression of uPA and c-met decreasing migration and proliferation abilities in HCCcells	Kutay et al., 2006; Huang et al., 2008; Salvi et al., 2009
miR-24	Promote cell growth and inhibit apoptosis	Kutay et al., 2006; Huang et al., 2008, 2009
miR-25	Apoptosis inhibition	Li et al., 2008, 2009c; Wang et al., 2008; Huang et al., 2009
miR-26a	Regulates the expression of cyclin D2 and E2 and induces G1 arrest of human liver cancer cells. Reduced expression in HCC. Systemic administration inhibe cancer cell proliferation and induced apoptosis in HCC	Chang et al., 2008; Ji et al., 2009a; Braconi et al., 2011; Kerr et al., 2011; Szabo et al., 2012
miR-27a	Promote cell growth and inhibit apoptosis	Huang et al., 2008, 2009
miR-29c	Apoptosis inhibition	Li et al., 2008; Xiong et al., 2010; Wang et al., 2011
miR-34a	Stimulation of HCC proliferation. Targeted c-Met and reduced both mRNA and protein levels of c-Met, thus blocking cell migration. Reduce invasion	Meng et al., 2007; Pogribny et al., 2007; Budhu et al., 2008; Gramantieri et al., 2008; Li et al., 2008, 2009a; Wong et al., 2008; Chen, 2009; Pineau et al., 2010
miR-92	The physiological significance of deregulation is still unknown	Meng et al., 2007; Shigoka et al., 2010
miR-93	Prevention of E2F1 acumulation. Proliferation	Kutay et al., 2006; Wong et al., 2008; Li et al., 2009c; Su et al., 2009; Pineau et al., 2010
miR-101/b-2	Downstream target of v-fos oncogene. Apoptosis inhibition. Inhibits cell proliferation and colony formation. Inhibits invasion and migration	Kutay et al., 2006; Gramantieri et al., 2008; Jiang et al., 2008; Li et al., 2008, 2009b; Su et al., 2009
miR-106a/b	Prevention of E2F1 acumulation. Proliferation	Kutay et al., 2006; Pogribny et al., 2007; Shah et al., 2007; Jiang et al., 2008; Ji et al., 2009a; Li et al., 2009c; Pineau et al., 2010
miR-122/a	Stimulation of HCC proliferation. Enhanced cell cycle progression. Modulates cyclin G1, influences p53 protein stability, and transcriptional activity and reduces migration and invasion capability of HCC-derived cell lines. Also Bcl-w is its direct target. Apoptosis inhibition	Kutay et al., 2006; Gramantieri et al., 2007; Meng et al., 2007; Budhu et al., 2008; Ladeiro et al., 2008; Lin et al., 2008; Wong et al., 2008; Bai et al., 2009; Chen, 2009; Coulouarn et al., 2009; Fornari et al., 2009; Huang et al., 2009; Liu et al., 2009; Tsai et al., 2009; Fan et al., 2011; Kerr et al., 2011; Li et al., 2011; Qi et al., 2011; Xu et al., 2012; Karakatsanis et al., 2013
miR-124/a-2	Stimulation of EMT. Supress cell proliferation	Gramantieri et al., 2007, 2008; Budhu et al., 2008; Huang et al., 2009; Furuta et al., 2010; Lang and Ling, 2012; Zheng et al., 2012a
miR-125a/b/b-2	Inversely correlated with aggressiveness and poor prognosis. Ectopic expression can inhibit the proliferation, invasion, and metastasis	Murakami et al., 2006; Meng et al., 2007; Budhu et al., 2008; Gramantieri et al., 2008; Li et al., 2008; Wong et al., 2008, 2010; Huang et al., 2009; Su et al., 2009; Kerr et al., 2011; Bi et al., 2012
miR-130/a/a-1/b	It is still unknown if contribute to HCC development and tumor progression	Kutay et al., 2006; Gramantieri et al., 2007; Jiang et al., 2008; Wong et al., 2008; Liu et al., 2009, 2012
miR-143	Promotes cancer cell invasion/migration and tumor metastasis by repression of fibronectin type III domain containing 3B (FNDC3B) expression	Gramantieri et al., 2007, 2008; Huang et al., 2009; Zhang et al., 2009
miR-145	Invasion and development	Gramantieri et al., 2007, 2008; Varnholt et al., 2008; Wang et al., 2008; Wong et al., 2008, 2010; Huang et al., 2009; Liu et al., 2009; Borel et al., 2012b; Karakatsanis et al., 2013
miR-146	Promote cell growth	Gramantieri et al., 2007; Xu et al., 2008; Karakatsanis et al., 2013
miR-148a/b	Metastasis	Budhu et al., 2008; Li et al., 2008, 2009b; Wong et al., 2008
miR-150	Cell differentiation and survival	Gramantieri et al., 2007, 2009; Jiang et al., 2008; Fornari et al., 2009; Zhang et al., 2009, 2012; Pineau et al., 2010
miR-151	Migration and invasion	Wang et al., 2008; Wong et al., 2008; Ding et al., 2010
miR-155	Development and invasion	Gramantieri et al., 2007; Wang et al., 2008; Huang et al., 2012
miR-181a/a-1/a-2/b/c/d	Migration. Enhanced MMP2 and MMP9	Gramantieri et al., 2007; Meng et al., 2007; Li et al., 2008; Garofalo et al., 2009; Ji et al., 2009a; Pogribny et al., 2009; Wang et al., 2010; Song et al., 2013
miR-182	Metastasis	Wang et al., 2008, 2012b; Wong et al., 2008, 2010
miR-183	Onset and progression, Apoptosis	Wang et al., 2008; Wong et al., 2008, 2010; Liang et al., 2013
miR-185	Metastasis	Budhu et al., 2008; Wong et al., 2008, 2010; Huang et al., 2009; Zhi et al., 2013
miR-192	Inhibition of DNA excision repair	Xie et al., 2011
miR-194	Metastasis	Budhu et al., 2008; Huang et al., 2009; Meng et al., 2010; Xu et al., 2013
miR-195	Proliferation, colony formation. Repressing Rb-E2F signaling. Enhanced G1-S transition	Murakami et al., 2006; Gramantieri et al., 2007, 2008; Wong et al., 2008, 2010; Huang et al., 2009; Liu et al., 2009; Xu et al., 2009
miR-199a/a^*^/a-1/a-2/b/-3p/-5p	MET, the tyrosine kinase HGF receptor, is post-transcriptionally regulated	Murakami et al., 2006; Gramantieri et al., 2007, 2008; Meng et al., 2007; Jiang et al., 2008; Wong et al., 2008, 2010; Chen, 2009; Liu et al., 2009; Su et al., 2009; Wang et al., 2009a; Kerr et al., 2011; Borel et al., 2012b
miR-200a/b/c	Stimulation of EMT	Murakami et al., 2006; Gramantieri et al., 2007; Jiang et al., 2008; Huang et al., 2009; Wong et al., 2010; Kim et al., 2011; Zhou et al., 2012; Karakatsanis et al., 2013
miR-203	Progression	Ladeiro et al., 2008; Chen et al., 2012b
miR-205	Proliferation	Huang et al., 2009; Wei et al., 2013a
miR-207	Metastasis	Budhu et al., 2008; Huang et al., 2009
miR-210	Metastasis	Meng et al., 2007; Wong et al., 2008; Su et al., 2009; Pineau et al., 2010; Ying et al., 2011
miR-214	Cell growth and invasion	Gramantieri et al., 2007; Jiang et al., 2008; Li et al., 2008; Wang et al., 2008; Wong et al., 2008, 2010
miR-221	Proliferation, colony formation, apoptosis, migration. Down-regulation of p27 and p57. Involved in the modulation of CDKN1B/p27 and CDKN1 C/p57, cell cycle proteina, and Bmf, a proapoptotic BH3-only protein. Promote cell cycle progression, reduce cell death and favor angiogenesis and invasion. TSC1/2 complex inhibition and enhanced AKT pathway. Enhanced MMP2 and MMP9. Inhibition of caspases 3, 6, 7, and 8	Volinia et al., 2006; Gramantieri et al., 2007, 2008, 2009; Meng et al., 2007; Fornari et al., 2008; Jiang et al., 2008; Li et al., 2008, 2011; Wang et al., 2008; Wong et al., 2008; Chen, 2009; Garofalo et al., 2009; Huang et al., 2009; Liu et al., 2009; Pogribny et al., 2009; Pineau et al., 2010; Wang et al., 2010; Kerr et al., 2011; Karakatsanis et al., 2013
miR-222	Enhanced AKT pathway. Enhanced MMP2 and MMP9. Inhibition of caspases 3, 6, 7, and 8. Migration, invasion	Gramantieri et al., 2007; Meng et al., 2007; Ladeiro et al., 2008; Li et al., 2008; Wang et al., 2008, 2010; Wong et al., 2008, 2010; Garofalo et al., 2009; Huang et al., 2009; Liu et al., 2009; Pogribny et al., 2009; Su et al., 2009; Pineau et al., 2010; Karakatsanis et al., 2013
miR-223	Proliferation. Inhibit cell viability. Inverse relationship with its downstream target Stathmin 1. Microtubules stabilization (G1-M transition)	Gramantieri et al., 2007, 2008; Jiang et al., 2008; Wong et al., 2008; Liu et al., 2009; Xu et al., 2011; Karakatsanis et al., 2013
miR-224	Promotes proliferation and inhibits apoptosis inhibitor-5 (API-5) transcript expression	Murakami et al., 2006; Meng et al., 2007; Gramantieri et al., 2008; Ladeiro et al., 2008; Li et al., 2008; Wang et al., 2008; Chen, 2009; Huang et al., 2009; Liu et al., 2009; Su et al., 2009; Pineau et al., 2010; Wong et al., 2010
miR-296-5p	It is still unknown if contribute to HCC development and tumor progression	Borel et al., 2012b; Katayama et al., 2012; Vaira et al., 2012; Wei et al., 2013b
miR-338/-3p	Associated with clinical HCC aggressiveness. Stimulation of HCC proliferation	Budhu et al., 2008; Gramantieri et al., 2008; Huang et al., 2009, 2011
miR-373	Invasion and metastasis	Meng et al., 2007; Bartels and Tsongalis, 2009; Wu et al., 2011
miR-374	Development	Wang et al., 2008; Wong et al., 2008, 2010; Koh et al., 2013
miR-375	Stimulation of HCC proliferation	Liu et al., 2010; He et al., 2012
miR-376a	Proliferation and apoptosis	Meng et al., 2007; Zheng et al., 2012b
miR-423	Enhanced CDK2 activity	Lin et al., 2011
miR-491-5p	Inhibition of TNF-α-related apoptosis	Yoon et al., 2010
miR-500	Elevated in HCC, returned to physiologic level after surgical intervention	Yamamoto et al., 2009
miR-637	Active STAT3	Zhang et al., 2011
let-7a/a-1/a-2/b/c/d/e/f/f-2/g	Development. Enhanced HCC proliferation, colony formation, and cell migration	Gramantieri et al., 2007, 2008; Budhu et al., 2008; Li et al., 2008; Wong et al., 2008; Huang et al., 2009; Liu et al., 2009; Ji et al., 2010; Pineau et al., 2010; Kerr et al., 2011; Lan et al., 2011; Sukata et al., 2011; Zhou et al., 2012

*Potential biomarkers of aflatoxin exposure*.

Cellular miRNAs can be released into the circulation, and circulating miRNA levels are also affected in HCC. Circulating plasma miRNA signatures may provide a novel diagnosis method for early, pre-symptomatic HCC patients, and may prove useful as prognosis biomarkers (Borel et al., 2012a).

In HCC has been reported up-expression of miR-21, miR-221, miR-22, miR-15, miR-517a, and down-expression of miR-122, miR-29 family, miR-26a, miR-124, let-7 family members, and miR-199a/b-3p (Szabo et al., 2012). Other miR- reported as markers involved in HCC have been miR-15b, miR-16, miR-17-5p, miR-18, miR-18a, miR-20, miR-23b, miR-26a, miR-29, miR-34a, miR-92, miR-101, miR-106a, miR-125b, miR-130b, miR-143, miR-146a, miR-195, miR-203, miR-223, miR-224, miR-338, miR-378, miR-422b, miR-500 (Murakami et al., 2006; Budhu et al., 2008; Jiang et al., 2008; Chen, 2009; Zhang et al., 2009; Kerr et al., 2011; Qu et al., 2011; Liu et al., 2012; Singhal et al., 2012; Wong et al., 2013).

In a review, Gramantieri et al. (2008) show miRNAs aberrantly expressed in HCC compared to non-tumorous liver tissue (up-expression of miR-33, miR-130, miR-135a, miR-210, miR-213, miR-222, miR-331, miR-373, miR-376a, and down-expression of miR-130a, miR-132, miR-136, miR-139, miR-143, miR-145, miR-150, miR-200a, miR-200b, miR-214). However, specific markers and their reliability are currently lacking as occur with aflatoxin-exposure.

Singhal et al. (2012), review molecular and serum markers in HCC as predictive tools for prognosis and recurrence. Aberrant expression of miR-21 can contribute to HCC by modulating PTEN expression and PTEN-dependent pathways (Meng et al., 2007). A significantly high expression of miR-224 in HCC patients promotes proliferation and inhibits apoptosis inhibitor-5 (API-5) transcript expression (Wang et al., 2008). An inverse correlation between miR-221 and both CDKN1B/p27 and CDKN1 C/p57, suggested miR-221 oncogenic function in hepatocarcinogenesis (Fornari et al., 2008). Also miR-221 has been involved in the modulation of Bmf, a proapoptotic BH3-only protein, regulating the cell proliferation and apoptosis proteins (Gramantieri et al., 2009).

HCC cells showed highly deregulated miR-223 expression and a strong inverse relationship with its downstream target Stathmin 1 (Wong et al., 2008). Murakami et al. (2006) and Li et al. (2008), found that miR-125b that suppress the cell growth and phosphorylation of Akt to be a prognostic marker of HCC. miR-122 found up to 70% of total miRNA in the liver, modulates cyclin G1, thus influences p53 protein stability and transcriptional activity and reduces invasion capability of HCC-derived cell lines (Fornari et al., 2009). Bcl-w is a direct target of miR-122 that functions as an endogenous apoptosis regulator in these HCC-derived cell lines (Lin et al., 2008). miR-122 is under the transcriptional control of HNF1A, HNF3A and HNF3B and loss of miR-122 results in an increase of cell migration and invasion. From a clinical point of view, miR-122 can be used as a diagnostic and prognostic marker for HCC progression (Coulouarn et al., 2009).

In HCC cell line, miR-34a directly targeted c-Met and reduced both mRNA and protein levels of c-Met, thus blocking cell migration (Li et al., 2009a). miR-18a high expression in HCC tumors (Liu et al., 2009). miR-101 has a downstream target of v-fos oncogene and it is involved in cell invasion and migration in overexpressed HCC cell lines (Li et al., 2009b). Xu et al. (2009) show that miR-195 may block the G(1)/S transition by repressing Rb-E2F signaling through targeting multiple molecules, including cyclin D1, CDK6, and E2F3. Upregulation of miR-143 expression transcribed by NF-kappa B in HBV-HCC promotes cancer cell invasion/migration and tumor metastasis by repression of fibronectin type III domain containing 3B (FNDC3B) expression (Zhang et al., 2009).

The level of miR-338 expression can be associated with clinical aggressiveness of HCC (Huang et al., 2009). miR-23b can recognize target sites in the 3-UTR of uPA and of c-met mRNAs and translationally repress the expression of uPA and c-met decreasing migration and proliferation abilities in HCC cells (Salvi et al., 2009).

MiR-126 down-regulation has been suggested to be directly linked to alcohol-induced hepatocarcinogenesis (Morgan et al., 2004). Microarray profiling studies showed reduction in miRNAs expression specific of HCV and HBV-associated cases: down-regulation of miR-190, miR-134, and miR-151 occurs in HCV cases, and of miR-23a, miR-142-5p, miR-34c, in HBV cases (Ura et al., 2009). MiR-96 was reported to be distinctively upregulated in HBV-associated HCC (Ladeiro et al., 2008), whereas miR-193b upregulation has been found upon transfection of HCV genome (Braconi et al., 2010). As quoted above, up-regulation of miRNAs, including miR-17-92 cluster, miR-106a, and miR-34, occurs during tamoxifen-induced hepatocarcinogenesis in female rats (Pogribny et al., 2007), also long-term-administration of 2-AAF resulted in disruption of regulatory miR-34a-p53 feed-back loop (Pogribny et al., 2009). In mice administered a choline-deficient and amino acid-defined diet, in which steatohepatitis precedes HCC development, microarray analysis identified that miR-155 was consistently up-regulated (Wang et al., 2009b).

Ross et al. (2010) analyzed the miRNA expression levels in control and conazole-treated mice. Conazol exposure induced many more changes in miRNA expression. All but one of the altered miRNAs were downregulated compared to controls. The authors suggest that this pattern of the altered miRNA expression represents a signature for tumorigenic conazole exposure in mouse liver.

This newly emerging area of research should unravel novel biomarkers of diagnostic as well as prognostic value in HCC.

## microRNA and aflatoxin B1

Exposure to environmental carcinogens may affect miRNAs expression in liver cells. While this concept is largely acceptable in principle, the specific miRNAs that are deregulated by various toxic and/or carcinogenic agents are yet to be fully documented. What we know at best today is the end-point of the process: the miRNAs whose expression is altered in HCCs (Table 1). However, results from some reports suggest that changes in expression of miRNAs may occur early in the process (Jiang et al., 2008), and these changes may be related to specific etiological factors, such as AFB1. These still preliminary evidences suggest the possibility of using miRNAs as early markers for aflatoxins exposure.

Exposure to natural or chemical environmental agents contributes to HCC development (Wild, 2009). In this context, uncovering relationships between exposure to environmental carcinogens and expression of miRNAs may reveal practical and sensitive biomarkers of toxic exposures and/or carcinogenicity testing (Wang et al., 2009a). A few reports addressed this hypothesis and revealed the existence of differential miRNAs expression patterns in HCCs in accordance with specific risk factors suggesting that exposure to specific risk factors could be responsible for the appearance of characteristic pathogenetic miRNA signatures (Elamin et al., 2011).

Although the precise roles of miRNA in the response to xenobiotics, drugs and chemical toxicants, remain to be established, there is little doubt that miRNAs are important in the cellular and *in vivo* responses to xenobiotics (Taylor and Gant, 2008). At this time, no specific studies on the effect of AFB1 on miRNA expression have been reported.

The field of miRNA and toxicology, particularly as it pertains to AFB1 toxicological outcome, is still in its beginnings. Nonetheless, there seems to be an increasing interest among toxicologists trying to understand the contribution of miRNA in regulating various toxicological outcomes through regulation of gene expression. A number of questions need to be addressed, such as a global role of miRNAs in cellular toxicity and disease; how miRNA biogenesis and expression affect susceptibility/resistance to xenobiotic-induced toxicity or disease (Taylor and Gant, 2008); whether cellular miRNAs form a regulatory networks and how perturbations of such network can cause toxicity/disease including developmental toxicity; how miRNAs may regulate transgenerational toxicological response through epigenetic regulation of gene and genome expression; as well as whether homologous miRNAs can be identified in an animal species based on known miRNA species and their action in other species or even in plant kingdom (Choudhuri, 2010).

Until today specific microRNAs as markers for aflatoxin-exposure and their reliability are currently lacking. The following are some potential candidates based on their elucidated mechanisms of action.

The high expression of miR-122 in the liver appears to correlate with a central role in various functions of normal and diseased livers (Lewis and Jopling, 2010; Negrini et al., 2011). It provides a very attractive target for aflatoxins. Rather surprisingly, given the high intracellular levels and numerous targets of miR-122, inactivation of the miRNA does not have any apparent adverse effects on liver physiology. However, reduced miR-122 expression does show an association with hepatocellular carcinoma, and further work will be necessary. In HCC, miR-122 is downregulated in approximately 70% of cases, suggesting a tumor suppressor function for this miRNA (Bai et al., 2009; Fornari et al., 2009; Ma et al., 2010; Callegari et al., 2013). In addition, loss of miR-122 expression in patients with liver cancer is correlated with the presence of metastasis and a shorter time to recurrence (Coulouarn et al., 2009; Fornari et al., 2009; Tsai et al., 2009). The role of miR-122 in liver cancer has been demonstrated directly by the generation of miR-122 knockout mice (Hsu et al., 2012; Tsai et al., 2012) These mice were characterized by hepatic inflammation, fibrosis, and development of spontaneous tumors similar to HCC, demonstrating the tumor-suppressor function of this miRNA and its important role in liver metabolism and differentiation of hepatocytes (Jensen et al., 2003; Gramantieri et al., 2007; Lin et al., 2008; Bai et al., 2009; Fornari et al., 2009; Tsai et al., 2009; Callegari et al., 2013).

On the other hand, up-regulation of miR-221, may be involved from the very early stage of hepatocarcinogenesis, and expression of the miRNA may progressively increase during malignant transformation. Especially, high expression of miR-221 can be used to predict local recurrence of HCC, and fold changes in miR-221 less than 1 can be used as a predictive marker of metastasis after curative surgical resection in patients with HCC (Yoon et al., 2011). Thus, among the miRNAs that are upregulated in HCC, there is evidence in support of the tumor-promoting activity of miR-221. It is upregulated in 70–80% of HCC samples (Fornari et al., 2008). From a functional point of view, HCC cells overexpressing miR-221 show increased growth, proliferation, migration, and invasion capability (Fornari et al., 2008; Medina et al., 2008; Garofalo et al., 2009; Gramantieri et al., 2009; Pineau et al., 2010; Callegari et al., 2012). Additionally, high level of miR-221 positively correlated with cirrhosis, tumor size and tumor stage, and negatively correlated with overall survival. miR-221 serum level monitoring could be of clinical relevance as a potential diagnosis tool and biomarker of treatment efficacy. It remains to be established which miRNA can sensitively and reliably be correlated with the presence of HCC at early stages of disease development and prognosis (Borel et al., 2012a).

miR-429 expression increased AFB1-DNA adducts in the SMMC-7721 Cells. To explore the effects of miR-429 expression on AFB1-DNA formation, Huang et al. (2013), accomplished a toxin experiment of AFB1 in the SMMC-7721 cells transfected by different mimics. Results showed that group with overexpression of miR-429 had elevated levels of AFB1-DNA adducts compared with control group. MiR-429 is classified as a member of miR-200 family and may play an important role in tumor prognosis. Overexpression of miR-429 induces cell proliferation and inhibits cell apoptosis. On the contrary, the suppression of miR-429 expression hindered cell proliferation and promoted cell apoptosis. These data suggest that this microRNA plays an important role in liver tumorigenesis, and functionally acts as an oncogene in HCC. Increasing evidence has shown that the levels of AFB1-DNA adducts correlate with HCC risk and prognosis, whereas the formation process of AFB1-DNA adducts can be modified by some factors such as detoxifying enzymes and DNA repair enzymes (Long et al., 2006, 2011; Xia et al., 2013). Is possibly that miR-429 can target some detoxification enzyme genes and/or DNA repair genes and reduce their detoxification capacity or DNA repair capacity and subsequently increase DNA damage and promote AFB1-DNA adducts formation. These results provided new insights into the mechanism of HCC induced by AFB1 (Huang et al., 2013).

The maintenance of genomic integrity through efficient DNA repair is essential for propagation of cellular life (Natarajan and Palitti, 2008). Nucleotide excision repair (NER) is one of the most versatile DNA repair system for elimination of bulky DNA adducts caused by environmental agents (Nouspikel, 2009) as AFB1 and other carcinogens. A possibility is that AFB1 could interfere with cellular NER through the regulation of microRNAs. Several miRNAs involved in DNA repair have been identified (Crosby et al., 2009; Yan et al., 2010; Hu and Gatti, 2011). A recent study showed that miR-192 directly targets a NER-associated protein (Georges et al., 2008). A bioinformatic analysis of miRNAs which potentially played a role in NER, show that miR-192, was the most differentially upregulated miRNA. The expression of ERCC3 and ERCC4 were reduced when miR-192 was overexpressed. Also has been observed that the relative repair capacity of damage by HepG2 and HeLa cells was reduced (Xie et al., 2011). Since of AFB1 is an important risk factor of HCC and AFB1-DNA adducts are known to be repaired by NER, dietary AFB1 exposure could impaired NER mediated by miRNAs like miR-192.

miR-500 is an oncofetal miRNA, which is highly expressed in fetal liver, more than in adult normal liver, and aberrantly expressed in HCC. This miRNA was associated with liver maturation in a mouse model of liver development. Levels tended to be higher in HCC lines and tumor samples when compared with matched normal tissue. Importantly, significant difference in miR-500 expression was found between normal liver and liver cirrhosis samples, suggesting that miR-500 expression was upregulated during cirrhosis development. An increased amount of miR-500 was found in the sera of 3 out of 10 HCC patients, which means that liver cancer-specific miRNA such as miR-500 is circulating in the peripheral blood and can be a novel diagnostic marker. These results show that the miR-500 abundance profile in serum of the HCC patients might reflect physiological and/or pathological conditions. However, although results are promising for miRNA-based HCC screening, further validation is suggested (Yamamoto et al., 2009).

miR-148, another candidate. There are also reports suggesting that drug-metabolizing enzymes such as CYP family genes are targeted by certain miRNAs. The expression of drug- and xenobiotic-metabolizing enzymes and nuclear receptors and their regulation by miRNA could be important factors for the outcomes of toxicity (Yokoi and Nakajima, 2011). Members of the CYP family are the most important enzymes catalyzing the metabolism of xenobiotics including drugs, environmental chemicals, and carcinogens. The different profiles of the expression of P450 isoenzymes determine the amount of reactive intermediates formed and the resulting toxic response. P450s are also known to bioactivate many procarcinogens to their ultimate carcinogens as in the case of AFB1. Recently, some P450s and nuclear receptors have been found to be post-transcriptionally regulated by miRNAs. Aflatoxin B1 and G1 are known to be oxidized efficiently to genotoxic metabolite(s) by CYP3A (Shimada et al., 1989; Forrester et al., 1990), epoxidation of AFB1 is catalyzed by CYP1A2 and CYP3A4 in humans (Gallagher et al., 1994; Ueng et al., 1995). The role of miRNA in the regulation of the expression of CYP3A4 has been reported, Takagi et al. (2008), found that miR-148 modulated inducible and/or constitutive levels of CYP3A4 in human liver cancer.

miRNAs are important regulators for CYP3A. Among these differentially regulated miRNAs, miR-155 appears to be the most prominent regulator as it was significantly associated with lower hepatic CYP3A activity (Vuppalanchi et al., 2013). CYP3A4 is the most abundant hepatic and intestinal CYP enzyme in humans, contributing to the metabolism of various drugs (Gonzalez and Yu, 2006), as AFB1. Pan et al. (2009) suggest that intervention of miRNA pathways may modify CYP3A4 expression and alter CYP3A4-catalyzed drug activation. Of particular note, miR-148a has been shown to control post-transcriptional regulation of PXR and, consequently, affect the expression of CYP3A4 (Takagi et al., 2008). Another study suggests that miR-27a and miR-27b may target RXR and regulated of CYP3A4 transcriptional expression (Ji et al., 2009b). The results indicate that intervention of miRNA pathways can be translated into an altered sensitivity of cells to xenobiotics. These findings may provide increased understanding of the complex regulation of CYP3A4 expression, as well as determine the role of miRNAs in drug metabolism and disposition (Pan et al., 2009).

Aflatoxin B1 (AFB1) is carcinogenic due its potential in inducing the oxidative stress and distortion of the most antioxidant enzymes (Abdel-Wahhab et al., 2007; El-Agamy, 2010; Alm-Eldeen et al., 2013). Recently, the role of miRNAs in oxidative stress-mediated etiology is emerging. Dong et al. (2013) found that miR-214 directly bound to 3′-UTR of the GSR and POR genes, and repressed their endogenous expressions and activities. These findings suggested miR-214 mediating down-regulation of glutathione reductase and CYP oxidoreductase genes might play an important role in oxidative stress in live cells. Wang et al. (2008, 2013) reported that miR-214 is one of the most significantly downregulated miRNAs in HCC patients. Extensive research has suggested that continued oxidative stress is a common pathologic pathway for most chronic diseases including cancer, and liver diseases. Therefore, Dong et al. (2013) postulated that miR-214 could be a key post-transcriptional regulator in oxidative stress-mediated human diseases. This microRNA will be also an important molecule to study in oxidative stress induced by AFB1 in liver.

Future validation for all of these miRNAs will be needed to assess their prognostic significance. It is notable that only a few miRNA signatures could potentially be used for diagnosis and prognosis, and even for these there is still a long way to go before they can be used in clinics. To achieve this goal, the miRNA signatures need to be further validated with high accuracy in prospective studies (Ji and Wang, 2009).

## Future perspectives in toxicological research

There is a need for novel markers that would combine the less invasiveness of a blood test and serve as a reliable early detection method. miRNAs definitely have this potential because not only they can be detected in plasma, but their sensitivity and stability are suitable for a clinical setting. Depending on the method, as little as one copy can be detected. The discovery of circulating miRNAs offers interesting clinical perspectives but this field of research is quite recent and more work has to be done.

Recently, measurement of circulating miRNAs has shown promise in identification of new biomarkers of liver injury. Further studies are needed to evaluate the sensitivity and specificity as well as validate the omics biomarkers of hepatotoxicity-xenobiotic exposure related.

It is difficult to establish the precise cause-effect relationships among environmental chemicals, miRNA alterations, and diseases. Future studies will need to demonstrate the contribution of environment-miRNA interaction to environmental human disease. The rapidly growing evidence linking miRNAs and environmental chemical, coupled with the unique regulatory role of miRNAs in gene expression, makes miRNAs potential biomarkers for elucidating the mechanisms and developing more effective prevention strategies for environmental diseases (Hou et al., 2011).

Currently, over five billion people worldwide experience uncontrolled exposure to aflatoxin (Strosnider et al., 2006). What remains unknown is how many liver cancer cases can be attributed to this aflatoxin exposure worldwide. Recently Liu and Wu (2010) have developed a risk assessment for the contribution of aflatoxin to the global burden of HCC. Of the 550,000–600,000 new HCC cases worldwide per year, they estimate about 25,200–155,000 (4.6–28.2%) may be attributable to aflatoxin exposure alone. The broad range in the estimate reflects limitations in determining levels of aflatoxin exposures, uncertainties in the nature of the dose-response curve, uncertainties in the mode of interaction between aflatoxins and viruses, and incomplete data on the prevalence of HBV in different regions of the world. Data driven estimates of the noncarcinogenic health effects of aflatoxins in humans have not been undertaken (Kensler et al., 2011).

The understanding of miRNA biology has advanced greatly in recent years, and the continuous technological advances in accurate miRNA detection, prospect a very promising role for miRNAs as novel biomarkers of environmental chemical exposure-related diseases Identifying chemical-specific miRNAs will not only help our understanding of environmental disease, but may open the way to novel biomonitoring and preventive strategies. Therefore, it is critically important to be able to identify and validate miRNAs that can be induced by specific environmental chemicals and regulate gene expression (Hou et al., 2011).

Understanding the miRNAs roles in toxicological processes requires overall a toxicogenomic approach. On the other hand, miRNA profiling data looks promising as a tool to predict the potential toxicity of unknown compounds. Thus, miRNA signatures of a known toxic compound may include miRNAs related to cellular response to stress, xenobiotic metabolism, and/or DNA repair. These signatures derived from supervised classification algorithms may effectively identify potential toxic compounds. Several examples of miRNAs active in cellular stress as well as in interactions of a number of toxicants. miRNA profiling may lead to the discovery of miRNA exposure biomarkers, which might work as sentinel molecules to better predict both efficacy and safety. The miRNA field in toxicology is still in its early stages. However, progress is occurring at a fast pace and the numbers of publications featuring miRNAs are increasing. As the roles of miRNAs in cellular response to xenobiotic stress and the development of physiological changes and other toxicological phenomenon such susceptibility and resistance are gradually uncovered, the coming years promise to be full of exciting avenues of miRNA research in toxicogenomics (Lema and Cunningham, 2010).

Potential microRNAs that could serve as possible markers of HCC by exposure to aflatoxins are miR-27a, miR-27b, miR-122, miR-148, miR-155, miR-192, miR-214, miR-221, miR-429, and miR-500. Future studies should include some of these microRNAs and confirm their relationship with the induction of HCC due to aflatoxin exposure.

### Conflict of interest statement

The authors declare that the research was conducted in the absence of any commercial or financial relationships that could be construed as a potential conflict of interest.
